# Our New Associate Editor and Owner

**Published:** 1895-06

**Authors:** 


### EDITORIAL.

THE JOURNAL takes pleasure in announcing that it has added to its editorial staff, as a joint editor and owner, the name of Prof. H. D. Gill, Secretary of the Faculty and Professor of Theory and Practice in the New York College of Veterinary Surgeons. Dr. Gill's long connection with the teaching staff of the above college, his many years of active practice in connection with the college hospital and outside practice, his association with the Bureau of Animal Industry as an Inspector, his close affiliation with the United States Veterinary Medical Association, and his State and local veterinary associations, mean much to the readers of this JOURNAL, and will add much strength to the efforts now being put forth to make the JOURNAL the best publication of its kind in North America. We bespeak for him at the hands of the readers as warm a welcome as has been accorded him by the Editors on his acceptance of a place with us in the publication of this periodical.

